# Distinct ECG Phenotypes Identified in Hypertrophic Cardiomyopathy Using Machine Learning Associate With Arrhythmic Risk Markers

**DOI:** 10.3389/fphys.2018.00213

**Published:** 2018-03-13

**Authors:** Aurore Lyon, Rina Ariga, Ana Mincholé, Masliza Mahmod, Elizabeth Ormondroyd, Pablo Laguna, Nando de Freitas, Stefan Neubauer, Hugh Watkins, Blanca Rodriguez

**Affiliations:** ^1^Department of Computer Science, University of Oxford, Oxford, United Kingdom; ^2^Division of Cardiovascular Medicine, Radcliffe Department of Medicine, University of Oxford, Oxford, United Kingdom; ^3^Biomedical Signal Interpretation & Computational Simulation Group, CIBER-BBN, University of Zaragoza, Zaragoza, Spain

**Keywords:** hypertrophic cardiomyopathy, electrocardiography, e-cardiology, phenotyping, computational clustering

## Abstract

**Aims:** Ventricular arrhythmia triggers sudden cardiac death (SCD) in hypertrophic cardiomyopathy (HCM), yet electrophysiological biomarkers are not used for risk stratification. Our aim was to identify distinct HCM phenotypes based on ECG computational analysis, and characterize differences in clinical risk factors and anatomical differences using cardiac magnetic resonance (CMR) imaging.

**Methods:** High-fidelity 12-lead Holter ECGs from 85 HCM patients and 38 healthy volunteers were analyzed using mathematical modeling and computational clustering to identify phenotypic subgroups. Clinical features and the extent and distribution of hypertrophy assessed by CMR were evaluated in the subgroups.

**Results:** QRS morphology alone was crucial to identify three HCM phenotypes with very distinct QRS patterns. Group 1 (*n* = 44) showed normal QRS morphology, Group 2 (*n* = 19) showed short R and deep S waves in V4, and Group 3 (*n* = 22) exhibited short R and long S waves in V4-6, and left QRS axis deviation. However, no differences in arrhythmic risk or distribution of hypertrophy were observed between these groups. Including T wave biomarkers in the clustering, four HCM phenotypes were identified: Group 1A (*n* = 20), with primary repolarization abnormalities showing normal QRS yet inverted T waves, Group 1B (*n* = 24), with normal QRS morphology and upright T waves, and Group 2 and Group 3 remaining as before, with upright T waves. Group 1A patients, with normal QRS and inverted T wave, showed increased HCM Risk-SCD scores (1A: 4.0%, 1B: 1.8%, 2: 2.1%, 3: 2.5%, *p* = 0.0001), and a predominance of coexisting septal and apical hypertrophy (*p* < 0.0001). HCM patients in Groups 2 and 3 exhibited predominantly septal hypertrophy (85 and 90%, respectively).

**Conclusion:** HCM patients were classified in four subgroups with distinct ECG features. Patients with primary T wave inversion not secondary to QRS abnormalities had increased HCM Risk-SCD scores and coexisting septal and apical hypertrophy, suggesting that primary T wave inversion may increase SCD risk in HCM, rather than T wave inversion secondary to depolarization abnormalities. Computational ECG phenotyping provides insight into the underlying processes captured by the ECG and has the potential to be a novel and independent factor for risk stratification.

## Introduction

Hypertrophic cardiomyopathy (HCM) remains a common yet challenging genetic heart muscle disease due to its heterogeneous clinical course. Ventricular arrhythmias are a major cause of sudden cardiac death (SCD) in young people (Maron et al., 2000; Maron, 2002). Accurate identification of high risk patients is a clinical priority since implantable cardioverter-defibrillators (ICD) can successfully treat ventricular arrhythmias triggering SCD.

In HCM, both ionic remodeling (Passini et al., 2016) and structural abnormalities [hypertrophy (Spirito et al., 2000), myocyte disarray (Varnava et al., 2001b) and fibrosis (Adabag et al., 2008)] create a pro-arrhythmic substrate to different extents in specific patients. Previous studies have attempted to assess the electrophysiological signature of HCM by visually inspecting the standard 12-lead paper electrocardiogram (ECG). Abnormalities such as abnormal Q waves, wide and high amplitude QRS complexes, ST segment displacement as well as giant inverted T waves have been reported in HCM (Savage et al., 1978; Lakdawala et al., 2011). However, no single abnormality was shown to be characteristic of HCM patients (Maron et al., 1983) and it is unclear whether T wave inversion is secondary to abnormal depolarization or a consequence of abnormal repolarization dynamics and heterogeneity. Furthermore, previous studies including cohorts of high-risk HCM patients also failed to produce reliable stratification, finding no differences in ECG between patients with and without appropriate ICD shocks (Maron et al., 1982; Sherrid et al., 2009). Some studies for example reported TWI to be related to increase in SCD risk (Ostman-Smith et al., 2010) but others did not (Maron et al., 1982; Sherrid et al., 2009). This may be due to the limitations in the methodology and therefore, more sophisticated approaches and new knowledge are required to improve the information extracted from the ECG for HCM phenotyping.

In the absence of reliable ECG biomarkers, five conventional risk factors [non-sustained ventricular tachycardia (NSVT), unexplained syncope, family history of SCD, massive left ventricular hypertrophy (LVH) and abnormal exercise blood pressure response] provide clinical utility for predicting SCD in HCM (Maron et al., 2007; Christiaans et al., 2010). More recently, the prospectively validated HCM Risk-SCD prediction model (O'Mahony et al., 2014) recommended by the 2014 ESC guidelines (Elliott et al., 2014), has performed better than conventional risk factors albeit with limitations (Maron et al., 2015). However, neither method captures the degree of the underlying myocardial abnormalities which lead to arrhythmic risk.

Our aim was to identify discrete subgroups of HCM patients with differences in electrophysiological and structural phenotype using novel computational analysis of high fidelity 12-lead Holter ECGs, through combined machine learning and mathematical modeling. To this end, we first extracted morphology-based biomarkers from the QRS using a mathematical model based on Hermite functions. We then applied an unsupervised clustering approach to ECG-based biomarkers, to automatically identify the presence of different phenotypic subgroups in the HCM population. Cardiac magnetic resonance (CMR) imaging and arrhythmic risk markers were evaluated to further characterize the patient subgroups. The low incidence of SCD in HCM of <1% per year (O'Mahony et al., 2013), and our reliance on patients without comorbidities, precluded its use as an endpoint in this study. Instead, we provide a deeper characterization of HCM phenotypes capitalizing on recently acquired digital ECG in conjunction with CMR data which is unattainable in larger retrospective databases. We hypothesized that detailed quantification of QRS morphology and T wave abnormalities using high fidelity ECGs would identify important features of the underlying electrophysiological and anatomical substrate, to enable improved phenotypic characterization of HCM patients.

## Materials and methods

### Ethics and study population

This prospective study was approved by the National Research Ethics Committee (REC ref 12/LO/1979) and informed written consent was obtained from each participant. Eighty-five patients with HCM were recruited from the University of Oxford Inherited Cardiac Conditions clinic, John Radcliffe Hospital, Oxford, UK. HCM diagnosis was made by presence of a pathogenic mutation in a known sarcomeric gene or, in the absence of an identified mutation, HCM was defined as LVH (≥15 mm) not originating from another cause. Gene positive patients without hypertrophy (G+LVH-) were included in the study as a number of SCDs have been reported in this patient cohort (Varnava et al., 2001a; Pasquale et al., 2012) and the consideration of LVH alone has limitations (Sen-Chowdhry et al., 2016); of note, five of the G+LVH- subjects had abnormal ECGs with voltage criteria for LVH. Thirty-eight age- and gender-matched healthy volunteers were non-smokers without cardiovascular disease, hypertension, diabetes, or family history of cardiomyopathy or SCD.

**Figure 1:**
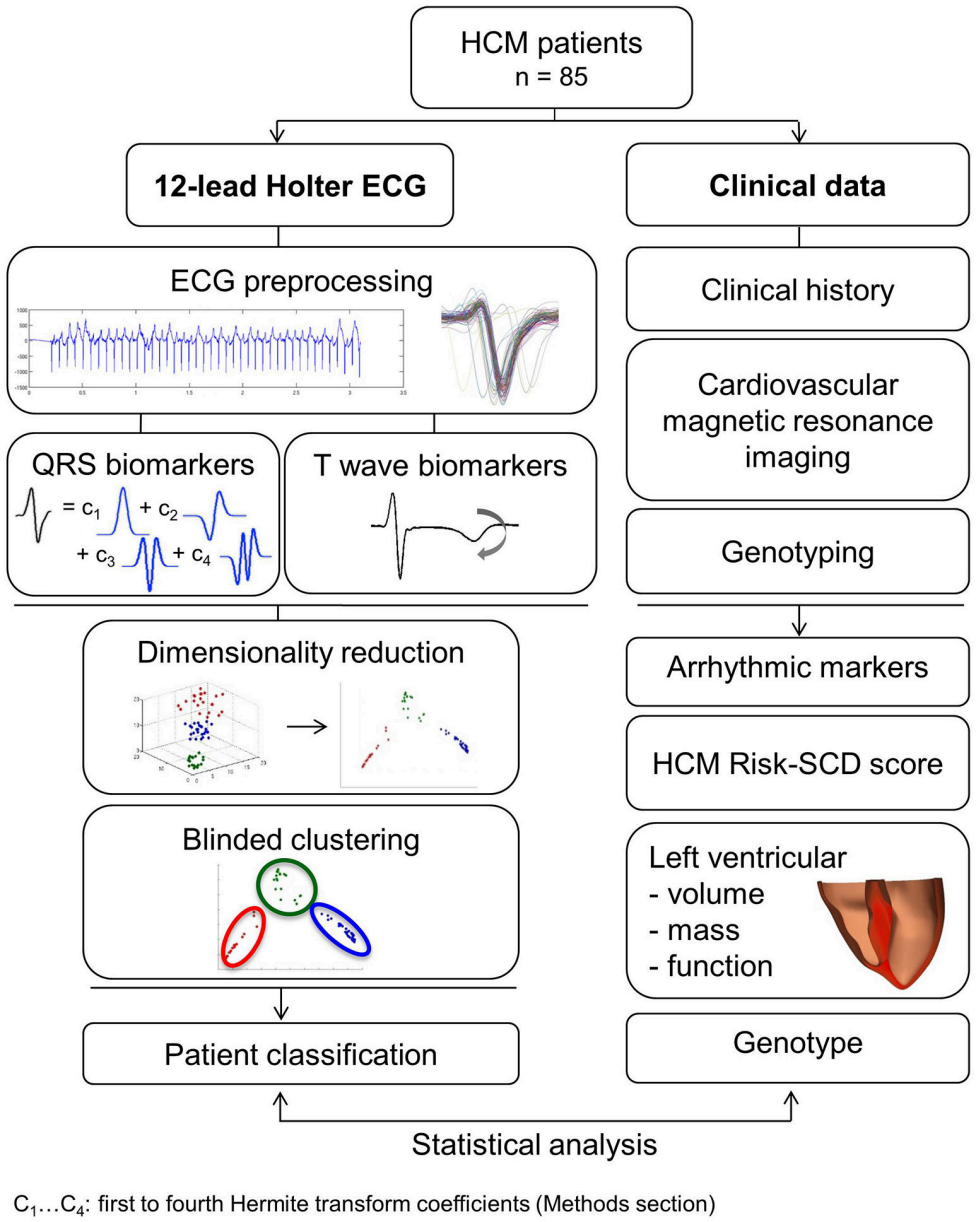
Summary of the methodologies applied in this study for the analysis and classification of 85 HCM patient ECGs using mathematical modeling and machine learning, and to investigate associations with clinical and cardiovascular magnetic resonance features.

The methodology is summarized in Figure 1 and detailed methods can be found in Supplementary Material 1.

### Electrocardiographic measurements

Participants underwent 12-lead ambulatory Holter monitoring (sampling frequency 1,000 Hz, H12+, Mortara Instrument, Milwaukee, WI, USA) for 24 h and a standard 12-lead resting digital electrocardiograph (Burdick 8500, Glasgow, UK) (Macfarlane et al., 1990). Standard measurements of ECG axes, amplitudes and intervals were obtained for both global (reported in Tables 1, **3**, **4**) and single-leads from the Burdick ECG XML files.

**Table 1:** Characteristics of healthy volunteers and HCM patients.

	**Healthy volunteers (*n* = 38)**	**HCM patients (*n* = 85)**	***p*-value**
Age, years	47 ± 15	45 ± 14	0.54
Male	25 (66)	58 (68)	0.84
Body mass index, kg/m^2^	24 ± 4	26 ± 4	0.06
Systolic BP, mmHg	117 ± 14	118 ± 14	0.76
Diastolic BP, mmHg	70 ± 10	72 ± 12	0.33
**CMR DIMENSIONS AND FUNCTION**			
LV end-diastolic volume, ml	155 ± 35	154 ± 33	0.88
LV end-systolic volume, ml	50 ± 17	41 ± 16	**0.007**
LV ejection fraction, %	68 ± 5	74 ± 7	**0.00003**
LV mass index, g/m^2^	54 ± 11	72 ± 25	**0.00002**
Maximal LV wall thickness, mm	11 ± 1	20 ± 6	**2** × **10**^−14^
Left atrial diameter, mm	33 ± 7	39 ± 7	**0.00004**
**ECG FEATURES**			
Heart rate, bpm	58 ± 11	58 ± 10	0.81
QRS axis, °	40 ± 34	13 ± 43	**0.0007**
QRS duration, ms	91 ± 9	98 ± 15	**0.02**
R wave amplitude	683 ± 200	764 ± 341	**0.46**
QRS amplitude, mV	1441 ± 414	1825 ± 652	**0.002**
QRS ascending slope	74 ± 22	89 ± 32	**0.025**
QRS descending slope	−118 ± 51	−150 ± 57	**0.001**
Pathological Q wave	0	20 (23)	**0.0004**
T wave axis, °	34 ± 17	69 ± 54	**0.0001**
Abnormal T wave axis	0	28 (35)	**9** × **10**^−6^
T wave amplitude, mV	356 ± 141	170 ± 257	**0.0001**
T wave inversion	0	26 (30)	**0.00002**
Giant T wave inversion	0	6 (7)	0.18
T peak to T end interval, ms	81 ± 14	85 ± 18	0.16
ST segment displacement, mV	42 ± 45	26 ± 49	0.14
QTc interval, ms	411 ± 17	440 ± 27	**8** × **10**^−8^
JTc interval, ms	300 ± 43	354 ± 99	**0.00003**

*Mean ± standard deviation, or number of participants (%). BP, blood pressure; CMR, cardiovascular magnetic resonance; LV, left ventricular. Bold values mean p-value significant (p < 0.05)*.

### Cardiovascular magnetic resonance (CMR) imaging

CMR imaging was performed at 3 tesla (TIM Trio, Siemens) in all participants, except in 10 patients with ICD at the time of enrollment (CMR prior to ICD insertion performed for clinical care was evaluated in these patients). Analysis was performed using cmr42© (Circle Cardiovascular Imaging, Calgary, Canada). The extent, and morphology, of hypertrophy was identified on short axis images and categorized into 4 subtypes: no hypertrophy—genotype positive patients with wall thickness ≤12 mm (G+LVH-); septal hypertrophy—basal and/or mid septal wall thickness >12 mm in genotype positive patients or ≥15 mm in genotype negative patients; apical hypertrophy—apical wall thickness ≥15 mm below papillary muscle level; mixed hypertrophy—coexisting hypertrophy in septal and apical segments.

### Clinical data collection

Genetic results and the conventional risk factors (Elliott et al., 2000) were obtained as part of the patient's routine clinical care. The five conventional risk factors were defined as: NSVT (three or more consecutive ventricular beats at a rate of ≥120 bpm lasting <30 s on clinical 3-lead 24- to 48-h Holter monitoring), unexplained syncope (≥1 episode of unexplained syncope), family history of SCD (history of SCD in ≥1 first degree relative ≤40 years old or SCD in a first degree relative with confirmed HCM at any age), massive LVH (LV wall thickness in any myocardial segment of ≥30 mm on short axis CMR images) and abnormal exercise blood pressure (BP) response (rise in systolic BP <20 mmHg or a fall of >10 mmHg from baseline to peak exercise in patients ≤40 years old). HCM Risk-SCD score (2014 ESC guidelines) was calculated using 7 disease variables as in O'Mahony et al. (2014) (Supplementary Material 1.4). A ≥6% risk of SCD at 5 years is classified as high risk and ICD implantation is recommended, 4–6% is intermediate risk and ICD may be considered, and <4% is low risk.

### Holter ECG pre-processing

The first 30-min ECG excerpt was used to analyze the 8 linearly independent leads (I, II, V1-6) with custom-built software using MATLAB (Mathworks, MA, USA). A wavelet based delineator (Martínez et al., 2004) extracted the peaks and boundaries of the ECG waveforms. High frequency noise was removed using a low-pass Butterworth filter with cut-off frequency of 45 Hz, baseline drift was removed by a cubic spline method and a notch filter rejected the 50 Hz mains power artifact. The first twenty beats with maximal ST segment-T wave signal-to-noise ratio were then considered for analysis and were aligned with respect to the QRS complex by Woody's method (Sörnmo and Laguna, 2005) (Supplementary Material 1.5). A sensitivity analysis showed that analyzing different 30-min excerpts in the recording yielded the same results. Thirty minutes also provided enough data to screen the excerpts and avoid changes in beat morphology due to changes in heart rate. Average QRS and STT waveforms were then computed from these 20 beats.

### Extraction of QRS and T wave biomarkers

All biomarkers were calculated per lead. QRS biomarkers are listed and illustrated in Supplementary Material 1.6, Figure S1. In addition to standard QRS biomarkers computed from signal processing, the QRS shape (morphology) was quantified by mathematically modeling the QRS waveform using a combination of Hermite functions, with well-established ability to provide a compact representation of the QRS complex (Laguna et al., 1996) (Supplementary Material 1.7, Figure S2). Indeed, three Hermite functions (Supplementary Material 1.7) enable to recover 98% of the QRS energy in control subjects (Sornmo et al., 1981). However, four Hermite functions were needed in HCM due to greater QRS heterogeneity. Any particular QRS morphology such as an RSR' pattern can thus be generated as the sum of these scaled shapes.

### Identification of subgroups in HCM using QRS and T wave biomarkers

Each patient was assigned a vector of morphological QRS and T wave biomarkers. Seven significant features were selected using the Multi-cluster feature selection method described in Cai et al. (2010), which is an unsupervised feature selection algorithm to reduce the number of variables under consideration. In short, the method consists in a combination of spectral analysis of the data with a L1-regularized least squares optimization method. This method was chosen for its ability to preserve the multi-cluster structure of the dataset. The 7 features were then reduced to two dimensions for visualization purposes, using Laplacian eigenmaps dimensionality reduction, as described in Belkin and Niyogi (2003). This method preserves the local geometrical properties of the dataset by computing the eigenvalues and eigenvectors of the graph Laplacian generalized eigenvector problem (Belkin and Niyogi, 2003).

A density-based clustering algorithm, DBSCAN (Ester et al., 1996), was then applied on this low-dimensional representation of the dataset to extract subgroups. This algorithm identified clusters by maximizing the density in each of the clusters. The minimum number of individuals in a cluster was set to *n*/25 = 3, with *n* = 86 HCM patients. The distance between neighboring individuals was evaluated using the Euclidean distance. The same results (i.e., same patients' subgroups) were obtained using a different clustering algorithm (k-means). Clustering analysis based only on QRS morphology was performed, and then repeated with the addition of T wave biomarkers. Clustering was performed by AL who was blinded to clinical data. To assess the effect of G+LVH- patients on the results, a further clustering analysis was performed excluding the G+LVH- patients.

### Statistical analysis

Data are expressed as mean ± standard deviation or median and range. Normally-distributed data were compared using *t*-tests or analysis of variance. Non-normally distributed data were compared using the Mann–Whitney *U*-test or Kruskal–Wallis test. Categorical data were compared with Chi-square or Fisher's exact tests. Statistical significance was assumed when *p* < 0.05 (after Bonferroni adjustment for multiple comparisons, where appropriate). Statistical analysis was performed with IBM SPSS Statistics, version 20.0 (IBM Corp, Armonk, NY, USA).

## Results

### Study population characteristics

The study population characteristics are summarized in Table 1. HCM patients (*n* = 85) had a more leftward QRS axis, larger QRS duration and amplitude, steeper QRS slopes and abnormal Q waves compared with healthy volunteers (*n* = 38) (all *p* < 0.03). They also showed lower T wave amplitude, T wave inversion (TWI), abnormal T wave axis and prolonged QTc (all *p* < 0.001). Table 2 describes the clinical characteristics for the HCM patients. Patients were mainly asymptomatic (median NYHA functional class = 1) and were low risk for SCD (median HCM Risk-SCD score = 2.5%; median total risk factor = 1). Nineteen patients had an ICD implanted for primary prevention with a median follow-up of 3 years. Only 1 patient (5%) received an appropriate shock, which is in keeping with primary prevention discharge rates seen in a previous study (Maron et al., 2000). Eleven percent of patients were G+LVH- and the majority had isolated septal hypertrophy (68%).

**Table 2:** Clinical and genotype characteristics of HCM patients.

NYHA class, [median (range)]	1 (1–3)
NYHA III/IV	5 (6)
LVOT obstruction (gradient ≥30 mmHg)	11 (13)
Implantable cardioverter-defibrillator	19 (22)
Appropriate ICD shocks	1
ICD follow-up, years [median (range)]	3 (0–12)
**HYPERTROPHY MORPHOLOGY**	
No LVH (G+LVH-)	9 (11)
Septal LVH	58 (68)
Apical LVH	4 (5)
Mixed septal & apical LVH	14 (16)
HCM Risk-SCD score, % [median (range)]	2.5 (0.8–11.0)
**CONVENTIONAL RISK FACTORS**	
NSVT	23 (27)
Syncope	10 (12)
Family History SCD	17 (20)
Abnormal exercise BP response	7 (8)
Massive LVH ≥30 mm	1 (1)
*Total number of risk factors [median (range)]*	1 (0–3)
*0 Risk factors*	34 (40)
*1 Risk factor*	45 (53)
*≥2 Risk factors*	6 (7)
**GENOTYPE**	
Gene negative	27(32)
MYBPC3	33 (39)
MYH7	24 (28)
Troponin I	1 (1)

*Number of patients (%). NYHA, New York Heart Association; LVOT, left ventricular outflow tract; ICD, implantable cardioverter-defibrillator; LVH, left ventricular hypertrophy; G+LVH-, genotype positive HCM with normal wall thickness; SCD, sudden cardiac death; NSVT, non-sustained ventricular tachycardia; BP, blood pressure; ESC, European Society of Cardiology; HCM, hypertrophic cardiomyopathy; MYBPC3, myosin-binding protein C; MYH7, beta-myosin heavy chain*.

### Clustering using QRS morphology only

**Figure 2:**
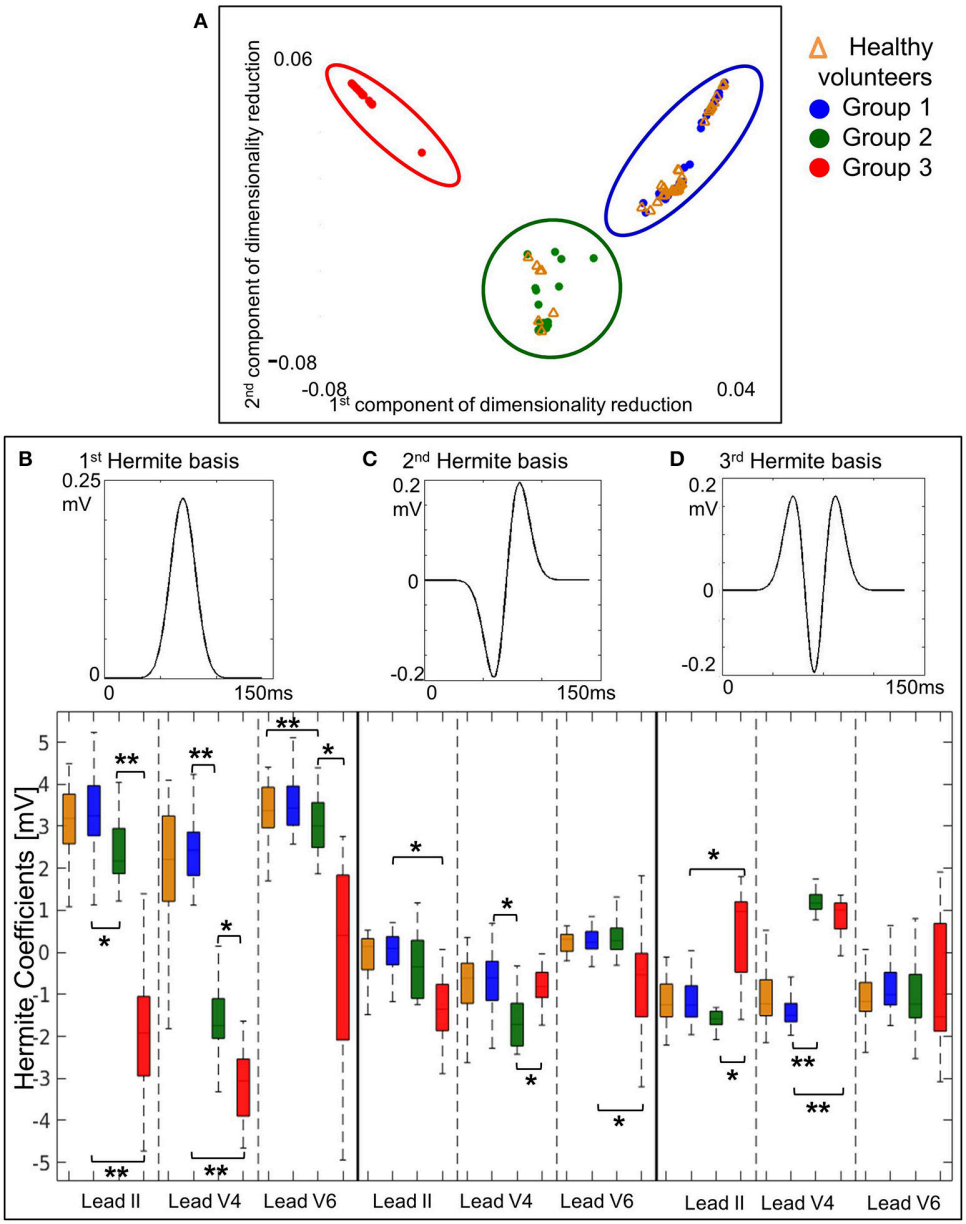
Clustering using QRS morphology alone identifies three HCM groups showing group differences in 3 features in lead II and the lateral precordial leads. **(A)** The three QRS-based HCM groups identified by cluster analysis using QRS morphological biomarkers alone are shown on the 2-dimensional space obtained by dimensionality reduction, as described in Materials and Methods section. **(B–D)** These QRS-based HCM groups show differences in the 1st, 2nd, and 3rd Hermite coefficients (mathematical functions representing the QRS shape: QRS morphological biomarkers) in leads II, V4 and V6. Healthy volunteers are shown for visual comparison but were not included in Kruskal–Wallis ANOVA (^**^p < 1 × 10^−6^, ^*^p < 0.001).

Based on QRS morphology alone, three HCM groups were obtained (Figure 2A), with the main differences in first, second and third Hermite basis in lead II and lateral precordial leads (Figures 2B–D; Supplementary Material 2.2, Figure S3). The ECG features for the QRS-based groups can be found in Supplementary Material 2.2, Table S1.

Group 1 was the largest with 52% of the patients, displaying normal QRS morphology (Figures 2, **4A**). Group 2 (22% of patients) showed differences in V4 (in the first three Hermite bases) compared to healthy volunteers and Group 1 but no difference in V6 (Figure 2, all *post-hoc p* < 0.001). Thus, lead V4 in Group 2 displayed shorter R wave duration (2: 38 ms, 1: 47 ms; 2 vs. 1, p = 0.003) and deeper S waves (2: −1,170 μv, 1: −568 μv; 2 vs. 1, *p* = 0.0003) compared to Group 1 (**Figure 4A**; Supplementary Material 2.2, Table S1).

Group 3 (26% of patients) exhibited vast differences in lead II and V4-6 (in the first three Hermite bases) compared to the other HCM groups and healthy volunteers (Figure 2, all *post-hoc* p < 6 × 10^−6^). Differences in lead II were manifested as abnormally shifted QRS axis toward left axis deviation (LAD) (3: −37°, 1: 30°, 2: 29°; 3 vs. 1 and 3 vs. 2, all *post-hoc p* < 2 × 10^−7^). In V4, the R wave duration was shorter than Group 1 and amplitude was shorter than Groups 1 and 2, S wave duration was longer than Group 1, and S amplitude was deeper than Group 1 (3 vs. 1 and 3 vs. 2, all *post-hoc p* < 0.003). In V6, R wave duration and amplitude were shorter, and S wave duration and amplitude larger than both the other groups (3 vs. 1 and 3 vs. 2, all *post-hoc p* < 0.01; **Figure 4A**).

Although ECG features were significantly different between the QRS-based groups, clinical features and markers of arrhythmic risk were not (Table 3), suggesting that QRS biomarkers alone, may not be useful for risk stratification.

**Table 3:** Clinical features for QRS-based HCM groups.

	**Group 1 (*n* = 44)**	**Group 2 (*n* = 19)**	**Group 3 (*n* = 22)**	***p*-value (group comparison)**
Age, years	47 ± 15	44 ± 12	43 ± 13	0.34
Male	30 (68)	12 (63)	16 (73)	0.79
Body mass index, kg/m^2^	27 ± 5	25 ± 4	25 ± 4	0.16
Systolic BP, mmHg	120 ± 13	117 ± 13	114 ± 15	0.28
Diastolic BP, mmHg	74 ± 11	73 ± 13	68 ± 12	0.13
HCM Risk-SCD score, %,	2.6 (1–11)	2.1 (1–6)	2.5 (1–9)	0.98
**CONVENTIONAL RISK FACTORS**				
NSVT	14 (32)	4 (21)	5 (23)	0.69
Syncope	6 (14)	2 (11)	2 (9)	0.91
Family History SCD	7 (16)	5 (26)	5 (23)	0.60
Abnormal exercise BPR	5 (11)	0	2 (9)	0.44
Massive LVH ≥30 mm	0	0	1 (5)	–
**HYPERTROPHY**				
LV mass index, g/m^2^	74 ± 28	63 ± 17	73 ± 26	0.30
Max LV wall, mm	19 ± 6	19 ± 5	21 ± 6	0.22
Hypertrophy morphology				**0.006**
No LVH (G+LVH-)	8 (18)	1 (5)	0	
Septal LVH	23 (51)	16 (85)	20 (90)	
Apical LVH	2 (4)	1 (5)	1 (5)	
Mixed septal & apical LVH	12 (27)	1 (5)	1 (5)	
**OTHER CLINICAL FEATURES**				
LV end-diastolic volume, ml	152 ± 28	155 ± 33	156 ± 41	0.87
LV end-systolic volume ml	40 ± 14	43 ± 14	41 ± 21	0.50
LV ejection fraction, %	74 ± 8	72 ± 8	74 ± 7	0.61
Left atrial diameter, mm	40 ± 7	37 ± 6	38 ± 8	0.25
LVOT gradient, mmHg	7.0 (4–111)	6.7 (2–110)	6.8 (3–92)	0.92
**GENOTYPE**				
Gene positive	28 (64)	13 (68)	17 (77)	0.59

*Mean ± standard deviation, median (range) or number of patients (%). HCM, hypertrophic cardiomyopathy; SCD, sudden cardiac death; NSVT, non-sustained ventricular tachycardia; BP, blood pressure; BPR, blood pressure response; LVH, left ventricular hypertrophy; LV, left ventricular; G+LVH-, genotype positive HCM with normal wall thickness; LVOT, left ventricular outflow tract. Bold values mean p-value significant (p < 0.05)*.

### Combined clustering with QRS morphology and T wave biomarkers identify four HCM phenotypes

**Figure 3:**
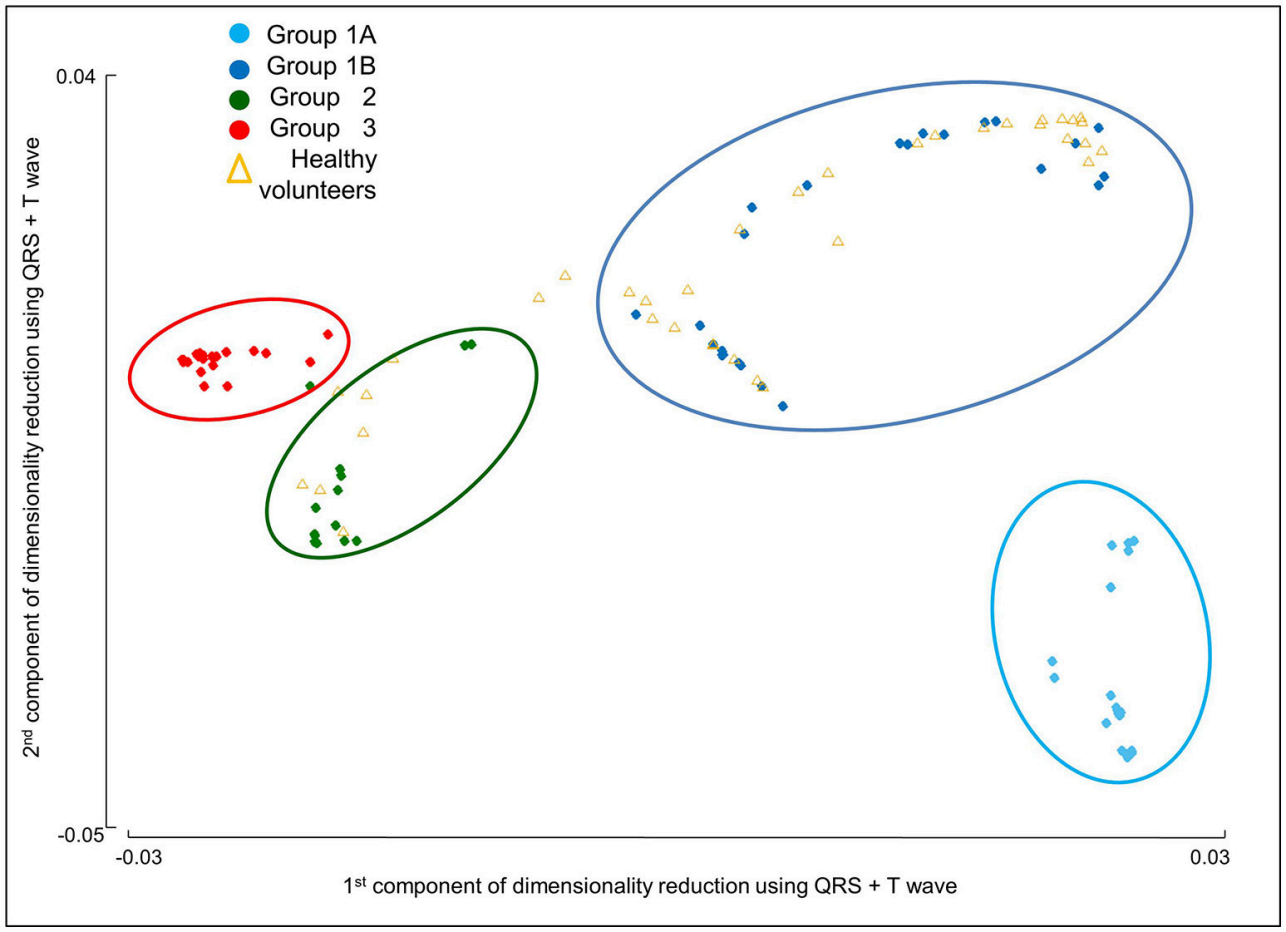
Four distinct HCM subgroups are identified based on QRS and T wave morphologies. The four HCM groups identified by cluster analysis using both QRS and T wave morphological biomarkers are shown on the 2-dimensional space obtained by dimensionality reduction, as described in Materials and Methods section.

**Figure 4:**
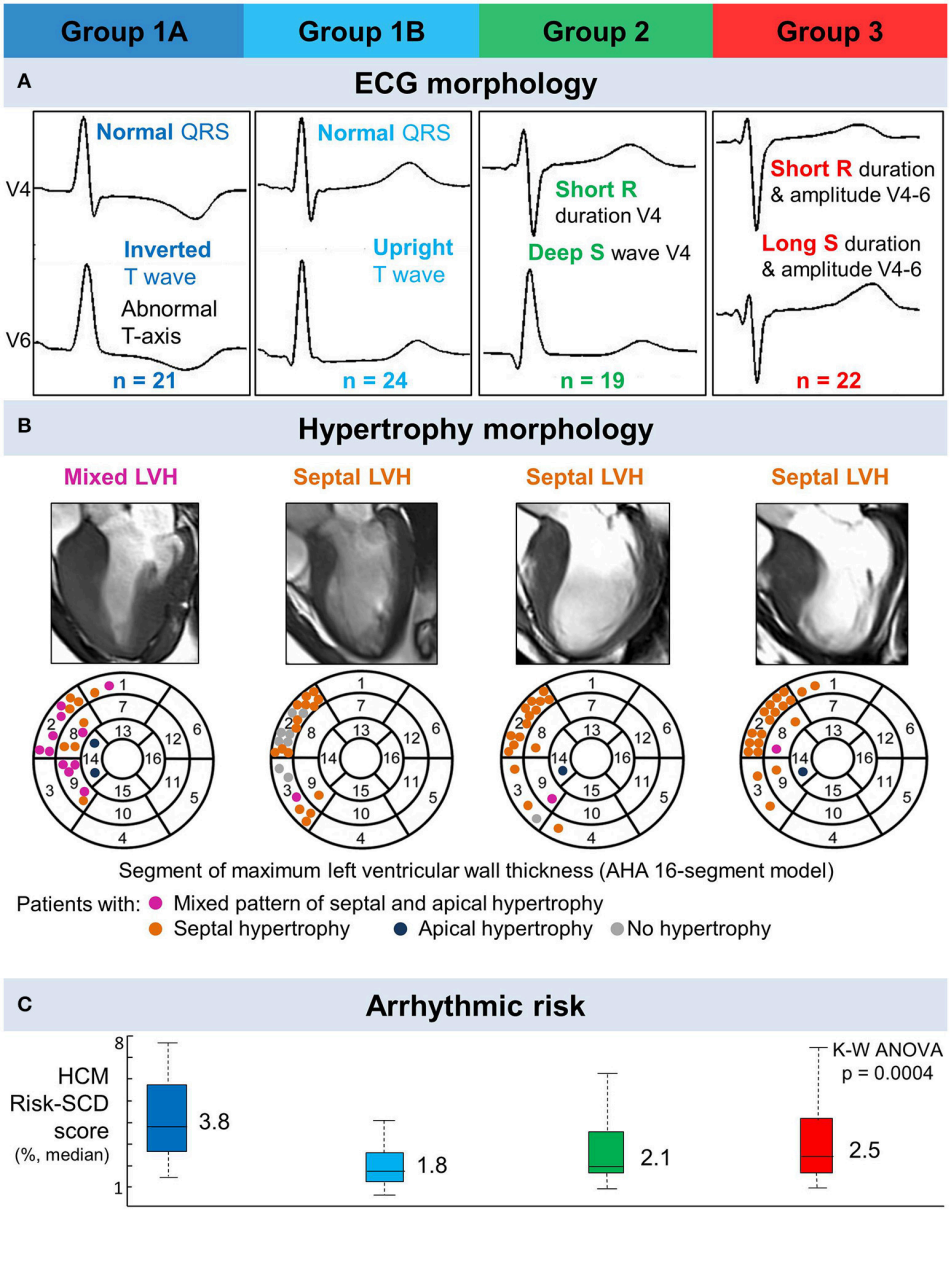
Four distinct ECG phenotypes in hypertrophic cardiomyopathy exhibit differences in hypertrophy morphology and arrhythmic risk. **(A)** Representative ECGs for patients in each of the four groups with distinct ECG morphology, identified by combined clustering with QRS morphology and T wave biomarkers. Group 1A—normal QRS with inverted T wave (primary T wave inversion), Group 1B—normal QRS with upright T wave, Group 2—short R wave duration and deep S wave in V4, Group 3—left axis deviation, short R wave duration and amplitude, and long S wave duration and amplitude in V4 and V6. **(B)** Distribution of hypertrophy illustrated using a representative CMR for each group (top), and the segment of maximum left ventricular wall thickness for each patient (marked as a dot) in each group using the AHA 16-segment model (Cerqueira et al., 2002). Group 1A had a predominance of patients with mixed septal and apical left ventricular hypertrophy (LVH; pink dots). Group 1B had the most gene positive patients with no hypertrophy (gray dots). Group 2 and 3 patients mainly had isolated septal hypertrophy (orange dots). Four patients had apical hypertrophy (navy dots). **(C)** HCM Risk-SCD score for each group. Patients with primary T wave inversion not secondary to QRS abnormalities (Group 1A), had the greatest HCM Risk-SCD score.

With the addition of T wave biomarkers to QRS morphology in the clustering analysis, four HCM groups were identified (Figure 3). TWI of the averaged beat in at least two contiguous leads in V3-6 was the principal T wave biomarker which subdivided Group 1 into two separate clusters. Groups 2 and 3 remained unchanged. Group 1A (*n* = 20) had normal QRS with TWI. Group 1B (*n* = 24) had normal QRS without TWI. Group 2 (*n* = 19) had short R wave duration and deep S wave amplitude in V4. Group 3 (*n* = 22) exhibited short R wave duration and amplitude together with long S wave duration and amplitude in V4-6, and left QRS axis deviation (Figure 4A; Supplementary Material 2.3). Table 4 summarizes the clinical and standard ECG features for the four HCM groups. Of note, Group 1B had an absence of patients with pathological Q waves (*post-hoc p* < 0.0005). There was no difference in demographics, LV volumes or genotype between the groups.

**Table 4:** Characteristics of the four HCM phenotypes from combined QRS and T wave clustering.

	**Group 1A (*n* = 20)**	**Group 1B (*n* = 24)**	**Group 2 (*n* = 19)**	**Group 3 (*n* = 22)**	***p*-value (group comparison)**
Age, years	47 ± 12	48 ± 18	44 ± 12	43 ± 13	0.53
Male	15 (76)	15 (63)	12 (63)	16 (73)	0.78
Body mass index, kg/m^2^	28 ± 6	26 ± 4	25 ± 4	25 ± 4	0.06
HCM Risk-SCD score, %	4.0 (2–11)^a^^,^^b^^,^^c^	1.8 (1–4)	2.1 (1–6)	2.5 (1–9)	**0.0001**
**CONVENTIONAL RISK FACTORS**					
NSVT	11 (55)^*^	3 (13)	4 (21)	5 (23)	**0.016**
Syncope	2 (10)	4 (17)	2 (10)	2 (9)	0.88
Family History SCD	5 (254)	2 (8)	5 (26)	5 (23)	0.36
Abnormal exercise BPR	2 (10)	3 (12)	0	2 (9)	0.51
Massive LVH ≥30mm	0	0	0	1 (5)	-
Appropriate ICD shock	1	0	0	0	-
**HYPERTROPHY**					
LV mass index, g/m^2^	90 ± 27^a^^,^^b^	62 ± 23	63 ± 17	73 ± 26	**0.0003**
Max LV wall, mm	22 ± 4^a^	16 ± 5	19 ± 5	21 ± 6	**0.002**
Hypertrophy morphology					**1** × **10**^−7^
No LVH (G+LVH-)	0	8 (33)^*^	1 (5)	0	
Septal LVH	7 (35)^*^	15 (63)	16 (85)	20 (90)	
Apical LVH	2 (10)	0	1 (5)	1 (5)	
Mixed septal & apical LVH	11 (52)^*^	1 (4)	1 (5)	1 (5)	
**OTHER CLINICAL FEATURES**					
LV EDV, ml	150 ± 26	154 ± 31	155 ± 33	156 ± 41	0.94
LV ESV, ml	35 ± 10	43 ± 15	43 ± 14	41 ± 21	0.26
LV ejection fraction, %	76 ± 7	72 ± 8	72 ± 8	74 ± 7	0.28
Left atrial diameter, mm	41 ± 5	39 ± 8	37 ± 6	38 ± 8	0.25
LVOT gradient, mmHg	6.5 (5–110)	7.2 (4–111)	6.7 (2–110)	6.8 (3–92)	0.81
**GENOTYPE**					
Gene positive	9 (45)	19 (79)	13 (68)	17 (77)	0.08
**ECG FEATURES**					
Heart rate, bpm	58 ± 10	58 ± 8	59 ± 13	58 ± 10	0.66
QRS axis, °	14 ± 30^c^	44 ± 29^e^	29 ± 34^f^	−37 ± 28	**8** × **10**^−13^
QRS duration, ms	94 ± 8	100 ± 21	96 ± 16	102 ± 13	0.23
QRS amplitude, mV	2006 ± 780	1762 ± 621	1807 ± 588	1737 ± 613	0.70
QRS ascending slope	93 ± 36	95 ± 33	92 ± 34	76 ± 23	0.19
QRS descending slope	−160 ± 60	−139 ± 56	−155 ± 60	−149 ± 56	0.47
Pathological Q waves	3 (14)	0^†^	8 (38)	9 (43)	**0.0003**
T wave axis, °	156 ± 45^a^^,^^b^^,^^c^	42 ± 27	49 ± 47	68 ± 41	**2** × **10**^−11^
Abnormal T axis	17 (81)^†^	0^†^	6 (33)	5 (25)	**7** × **10**^−8^
T amplitude, mV	−135 ± 202^a^^,^^b^^,^^c^	308 ± 144	219 ± 209	257 ± 214	**9** × **10**^−8^
T wave inversion	21 (100)^†^	0	4 (21)	1 (5)	**8** × **10**^−17^
Giant T wave inversion	5 (24)^†^	0	1 (5)	0	**0.003**
T peak to T end, ms	91 ± 17	80 ± 20	86 ± 16	84 ± 19	0.19
ST segment displacement^*^, mV	−13 ± 51^a^^,^^b^^,^^c^	32 ± 33	38 ± 51	48 ± 38	**0.0001**
QTc interval, ms	452 ± 22	435 ± 26	429 ± 26	443 ± 29	0.05
JTc interval, ms	359 ± 47	349 ± 87	342 ± 97	366 ± 145	0.42

*Mean ± standard deviation, median (range) or number of patients (%). HCM, hypertrophic cardiomyopathy; SCD, sudden cardiac death; NSVT, non-sustained ventricular tachycardia; BPR, blood pressure response; LVH, left ventricular hypertrophy; LV, left ventricular; G+LVH-, genotype positive HCM with normal wall thickness; EDV, end-diastolic volume; ESV, end-systolic volume; LVOT, left ventricular outflow tract*.

a *Group 1A vs. 1B*,

b *1A vs. 2*,

c *1A vs. 3*,

d *1B vs. 2*,

e *1B vs. 3*,

f *2 vs. 3, p < 0.05 on post-hoc pairwise comparisons (p-values multiplied by 6 for Bonferroni adjustment of 6 tests)*.

*, † *p < 0.05 on post-hoc contingency table analysis (p-values multiplied by 16 and 8 for Bonferroni adjustment of 4 × 4 and 4 × 2 combinations, respectively). Bold values mean p-value significant (p < 0.05)*.

### Differences between the four HCM phenotypes in HCM risk-SCD score and extent of LV hypertrophy

Group 1A had the highest median HCM Risk-SCD score (1A: 4.0%, 1B: 1.8%, 2: 2.1%, 3: 2.5%; 1A vs. 1B, 1A vs. 2, 1A vs. 3, all *p* ≤ 0.02; Figure 4C). NSVT differed between the 4 phenotypes (*p* = 0.016) but this was not significant when corrected for the three other risk factors also tested. Group 1A also contained the only ICD patient with an appropriate shock. Group 1B contained 8 of the 9 G+LVH- patients (Table 4, *post-hoc p* = 0.0003), of which 5 patients demonstrated ECG voltage criteria for LVH. In spite of their more abnormal QRS morphology, Groups 2 and 3 patients had a lower HCM Risk-SCD score than Group 1A patients (3: 2.5%, 2: 2.1%, 1A: 3.8%; 3 vs. 1A, 2 vs. 1A; *post-hoc* ≤ 0.02).

Group 1A exhibited a predominance of patients with mixed septal and apical hypertrophy (1A: 11 patients, 1B: 1 patient, 2: 1 patient, 3: 1 patient; *post-hoc* p = 4 × 10^−6^; Figure 4B). Groups 1B, 2 and 3 had predominantly isolated septal hypertrophy (63, 85, and 90%, respectively).

We also evaluated the effect of excluding the 9 G+LVH-patients on the results (Supplementary Material 2.4, Table S2). The clustering algorithm yielded the same four remaining groups as with G+LVH-patients, with the same differences in QRS and T wave morphologies. Group 1A still exhibited a higher HCM Risk-SCD score compared to Group 1B and to Group 3 (*post-hoc* *p* = 0.006 and 0.04, respectively), and there was still a predominance of mixed septal and apical LVH in Group 1A.

## Discussion

The main findings of this study are that four HCM phenotypes are identified based on QRS morphology and T wave biomarkers analyzed computationally using high fidelity ECGs, and they show differences in HCM Risk-SCD score and the distribution of LV hypertrophy from CMR. Patients with normal QRS morphology and primary TWI not secondary to QRS abnormalities (Group 1A) had the highest HCM Risk-SCD score and coexistence of septal and apical hypertrophy. Groups 2 and 3 with abnormalities in QRS morphology in V4 and V4-V6, respectively, had predominantly isolated septal hypertrophy. Since the ECG reflects ionic and structural abnormalities which are not captured by current measures within HCM risk stratification, the ECG-based classification proposed here may help to improve risk assessment. Our study shows the benefits of using machine learning methods to effectively dissect HCM heterogeneity, and presents a step forward in improving individual patient management.

### QRS morphology

QRS morphology reflects the cardiac depolarization sequence and is therefore affected by structural abnormalities such as hypertrophy, cardiac disarray or fibrosis. Here we have quantitatively assessed the whole QRS shape (morphology) mathematically rather than describing discrete features. Cluster analysis with QRS morphology alone identified three groups with unique QRS features (Figure 2) but these QRS variations could not be accounted for by differences in hypertrophy. It is therefore likely that fibrosis (Dumont et al., 2006) and disarray affect depolarization and QRS particularly in patients in Groups 2 and 3. CMR studies using tissue characterization techniques such as late gadolinium enhancement (focal fibrosis), T1-mapping (diffuse fibrosis) and diffusion tensor imaging (disarray) are likely to provide further insights. Our analyses suggest that while QRS features may be informative for diagnosis in HCM (Konno et al., 2004), categorizing HCM groups on this basis showed no differences in association with known markers of risk.

### T wave inversion

Following the classification in three groups based on QRS morphology, lateral TWI, a common repolarization abnormality in HCM (Papadakis et al., 2009), as the critical T wave biomarker which separated patients with normal QRS into those with and without TWI. Groups with abnormalities in QRS were not affected by the inclusion of TWI in the clustering. Group 1A patients with TWI, even though with a normal QRS, had a higher HCM Risk-SCD score, an appropriate ICD shock and a predominance of mixed septal and apical pattern of hypertrophy. TWI can be considered as primary or secondary abnormalities. Primary TWI occurs with altered heterogeneity in action potential duration or morphology without changes in depolarization (normal QRS). Secondary TWI occurs with aberrant depolarization (abnormal QRS) in the context of normal action potential characteristics (Fisch, 1992). Thus Group 1A patients with normal QRS have primary. While TWI in Groups 2 and 3 with abnormal QRS represents secondary or combined primary and secondary TWI (Rautaharju et al., 2009). However only four patients in Group 2 and a single patient in Group 3 had TWI, while all 20 patients in Group 1A displayed TWI. TWI *per se* in HCM has been shown to increase SCD risk in some studies (Kuroda et al., 2002; Ostman-Smith et al., 2010) but not in others (Maron et al., 1982; Sherrid et al., 2009). Our results provide a more specific characterization of the influence of TWI in SCD risk, highlighting the importance of simultaneous normal QRS and TWI for increased risk. These results suggest that it is a primary TWI that increases SCD risk in HCM, rather than TWI secondary to depolarization abnormalities. A larger cohort is needed to confirm whether risk differs between patients with primary and secondary TWI.

TWI may be caused by repolarization abnormalities due to structural and ionic remodeling. In HCM, the overexpression of the L-type Ca^2+^ current, increased late sodium current and reduction of repolarization currents lead to prolongation and heterogeneity in repolarization (Coppini et al., 2013; Passini et al., 2016). TWI may also be the result of ischaemia from microvascular dysfunction commonly seen in HCM (Petersen et al., 2007). A study has shown that patients with apical HCM and cavity obliteration had increased perfusion defects and NSVT rates as a result of ischaemia from extravascular compression of the coronary artery due to myocardial pressure during cavity obliteration (Matsubara et al., 2003). This may also be in keeping with Group 1A having the greatest number of patients with a mixed pattern of coexisting septal and apical hypertrophy which tend to show cavity obliteration and a worse prognosis (Yan et al., 2012); while Group 3 with 90% septal hypertrophy, were at lower risk. Further imaging studies with novel perfusion assessment may improve our understanding of the mechanisms of ischaemia and in turn, the repolarization abnormalities in HCM.

### Low risk phenotypes

Patients in Group 1B with normal QRS morphology and upright T waves were indistinguishable from healthy volunteers based on the extracted ECG features both with and without the inclusion G+LVH- patients. Finding the majority of G+LVH- patients (8 out 9 patients) in Group 1B showed that we can discriminate these inherently low risk patients solely by the ECG, despite 5 of these patients meeting ECG voltage criteria for LVH. Furthermore, the presence of G+LVH- patients in Group 1B suggests that those patients with hypertrophy within this group are likely to have less severe disease and better prognosis (McLeod et al., 2009). We may also speculate that they will have minimal ionic remodeling, fibrosis, disarray and ischaemia giving rise to relatively normal depolarization and repolarization.

Despite the lack of hypertrophy, one G+LVH- patient was found in Group 2 which had QRS abnormalities in V4. This suggests that the ECG reflects the subtly abnormal myocardium which in this case was not hypertrophied, but may have been affected by disarray or ionic remodeling. Although risk is thought to be significantly lower in G+LVH- than in HCM with hypertrophy, there are still a very small number of SCD in G+LVH- patients (Varnava et al., 2001a; Pasquale et al., 2012). Further studies are needed to assess whether computational ECG phenotyping may aid stratification in this particularly challenging low-risk group.

Group 3 patients had marked QRS abnormalities: LAD with QRS differences in V4 to V6. LAD is well-known to be associated with LVH but other factors such as a degree of left anterior fascicular block could also account for this leftward axis. Group 3 patients mainly had isolated septal hypertrophy, yet QRS abnormalities were seen in the lateral leads suggesting that remodeling may occur distal from the septum causing less uniform electrical propagation in the lateral leads. No genotype association was seen across any group but our sample size may not have had adequate power to assess genotype-phenotype correlations.

### Clinical implications

This study provides evidence that ECG phenotyping with advanced computational QRS morphology and T wave analysis is a powerful method of characterizing HCM heterogeneity. Data suggest that HCM patients with a primary TWI (with normal QRS) are at greater risk of arrhythmia and SCD. This risk was associated with the distribution of hypertrophy (greater number of segments involved in mixed septal and apical pattern of hypertrophy) rather than magnitude of hypertrophy (as measured by maximum wall thickness or mass index). A large scale longitudinal study with cardiovascular end-point data will allow robust assessment of ECG phenotyping as an independent tool for accurate risk stratification. Studies involving computational image-based modeling and simulation are also needed to disentangle the relative contribution of structural, ischemic, and ionic factors which are likely to determine the heterogeneity in ECG biomarkers (Dutta et al., 2016). This improved understanding of HCM will eventually contribute to the development of new disease-modifying therapies.

### Limitations

Our study used digital ECG data from 12-lead Holter recorders. This enabled the identification of four distinct phenotypes using novel computational methods, which is not possible with standard paper ECGs collected in large studies. For these novel computational methods to be widely translated to clinical studies and practice, there needs to be drive toward digital ECG acquisition rather than paper print-outs, which require manual digitization before mathematical modeling and machine learning methods can be applied.

Given the large information content gathered for each patient in our study, the database is necessarily limited in the number of patients assembled. We included HCM patients without co-morbidities (described in Supplemental Material 1.1) to ensure there were no confounders in our data. Our analysis was however able to identify different patient subgroups and also with differences in risk. Over the next 5–10 years, a large prospective long-term follow-up study such as the multicenter Hypertrophic Cardiomyopathy Registry (HCMR) (2,750 patients) (Kramer et al., 2015) may provide the data to determine whether our findings allow improving current risk stratification for SCD using scanned paper 12 lead ECGs. Our study provides the detailed analysis based on high fidelity ECG recordings that would enable such validation. As a follow-up, a larger dataset would make possible to consider a supervised machine learning approach, such as support vector machines, random forests or neural networks (Lyon et al., 2018), taking as input both ECG biomarkers and risk scores to identify the subgroup at higher risk. It would also allow the use of more complex unsupervised approaches such as self-organizing networks, as proposed in Lagerholm et al. (2000). However, large databases usually do not include the comprehensive set of modalities we include in our study. For example, these big databases of 967 and 2,485 HCM patients (McLeod et al., 2009; Cortez et al., 2017) do not provide high-fidelity recordings and lack CMR data.

Limited accuracy of an ECG criterion can also result from variations in electrode placement especially in precordial electrodes (Kania et al., 2014). However, minimal changes in morphology were observed in leads V4-6. Therefore, criteria based on the lateral leads (which indeed demonstrated the greatest discrimination in HCM) would be robust to the inevitable variability of electrode site placement.

## Conclusions

Four HCM phenotypes were identified based on QRS morphology and T wave biomarkers using a machine learning approach. Patients with primary TWI not secondary to QRS abnormalities had an increased HCM Risk-SCD score and coexisting septal and apical hypertrophy. These results, and the nature of the underlying processes captured by the ECG, suggest that computational ECG phenotyping has the potential to be a novel and independent factor for risk stratification.

## Author contributions

RA recruited the HCM and Control populations and performed the statistical analysis; AL performed the ECG signal analysis and the computational clustering; RA and AL worked on the writing of the manuscript; MM and EO provided help in the collection of the data; PL and NdF gave input on the computational methods for signal processing and clustering; SN, HW, AM, and BR provided help and guidance on the study design and the writing of the manuscript.

### Conflict of interest statement

The authors declare that the research was conducted in the absence of any commercial or financial relationships that could be construed as a potential conflict of interest.

## Abbreviations

AHA: American Heart Association
BP: blood pressure
BPR: blood pressure response
CMR: cardiovascular magnetic resonance
ECG: electrocardiogram
G+LVH-: genotype positive HCM with normal left ventricular wall thickness
HCM: hypertrophic cardiomyopathy
ICD: implantable cardioverter-defibrillator
LAD: left axis deviation
LV: left ventricle
LVH: left ventricular hypertrophy
LVOT: left ventricular outflow tract
MYBPC3: myosin-binding protein C
MYH7: beta-myosin heavy chain
NSVT: non-sustained ventricular tachycardia
NYHA: New York Heart Association
SCD: sudden cardiac death
TWI: T wave inversion.
