# Supplementary material for: Distinct ECG Phenotypes Identified in Hypertrophic Cardiomyopathy Using Machine Learning Associate With Arrhythmic Risk Markers
Source: Front Physiol. 2018 Mar 13;9:213. doi: 10.3389/fphys.2018.00213 (PMC5859357; doi:10.3389/fphys.2018.00213)
Supplement: Supplementary file 1 [file DataSheet1.docx]

Supplementary Material

Distinct ECG phenotypes identified in hypertrophic cardiomyopathy using machine learning associate with arrhythmic risk markers

Aurore Lyon^1*^, Rina Ariga^2*^, Ana Minchole^1^, Masliza Mahmod^2^, Elizabeth Ormondroyd^2^, Pablo Laguna^3^, Nando de Freitas^1^, Stefan Neubauer^2^, Hugh Watkins^2^, Blanca Rodriguez^1^

^1^ Department of Computer Science, University of Oxford, Oxford, United Kingdom ^2^ Division of Cardiovascular Medicine, Radcliffe Department of Medicine, University of Oxford, Oxford, United Kingdom
^3^ Biomedical Signal Interpretation & Computational Simulation (BSICoS) Group, University of Zaragoza, CIBER-BBN, Spain

*** Equal contribution first authors**

**Correspondence:**Professor Blanca Rodriguez, PhD
blanca.rodriguez@cs.ox.ac.uk

# Methods

## Ethics and study population

Patients were excluded if they had a diagnosis of coronary artery disease, diabetes, hypertension or atrial fibrillation. ICD patients with a paced rhythm were also excluded due to the alterations in the ECG from pacing. Patients with no genetic mutation identified and with LVH underwent an echocardiogram as part of their diagnostic work-up having presented to a clinician with symptoms of shortness of breath, chest pain, palpitations or fainting. If the LV wall thickness measured ≥15mm with no other cause for hypertrophy, the patient was referred to the Inherited Cardiac Conditions clinic for specialist assessment. A genetic test was offered if the patient was thought to have HCM. Following this, we sought to enrol them in the study.

## Resting ECG recordings

Q waves were considered pathological if they were >30ms in duration and >1/3 of the R wave in depth in two or more contiguous leads (I, II, V1-6). Abnormal T wave axis deviation was classified as <-15º to ≥-180º or >105º to ≤180º (6 patients with complete bundle branch block; QRS duration ≥120ms, were excluded from T wave axis analysis due to secondary T wave changes from altered depolarization). T wave inversion was defined as T wave amplitude ≤ −0.1mV and giant T wave inversion ≤ −1.0mV in two or more contiguous leads in V3-6. The ECGs were performed by either a senior cardiologist (RA) or senior cardiac nurse (JS) according to the 2007 AHA/ACC guidelines on ECG Part 1. The Burdick recording was 10 seconds with 12 leads recording simultaneously. The sampling rate was 8000Hz.

## Cardiovascular Magnetic Resonance (CMR) imaging

Left ventricular (LV) volumes, mass and function was calculated from a steady state free precession sequence of contiguous LV short axis slices of 8mm thickness with an interslice gap of 2mm. Left atrial diameter was measured antero-posteriorly at end-systole in the 3-chamber left ventricular outflow tract view. The degree of LV hypertrophy was measured by two methods: maximal wall thickness – a focal measure used in SCD risk assessment, and mass index – a global measure.

## Clinical data collection

LV outflow tract (LVOT) gradient was measured at rest and/or on Valsalva provocation using transthoracic echocardiography.

HCM Risk-SCD score (2014 ESC guidelines) was calculated for each patient using 7 disease variables (1) in the following formula: Probability_SCD at 5 years_ = 1 − 0.998^exp(Prognostic index)^, where Prognostic index = [0.15939858 × maximal wall thickness (mm)] − [0.00294271 × maximal wall thickness^2^ (mm^2^)] + [0.0259082 × left atrial diameter (mm)] + [0.00446131 × maximal (rest/Valsalva) LVOT gradient (mmHg)] + [0.4583082 × family history of SCD] + [0.82639195 × NSVT] + [0.71650361 × unexplained syncope] - [0.01799934 × age at clinical evaluation (years)].

## Holter ECG pre-processing

The first twenty beats with maximal ST segment‒T wave (STT) signal-to-noise ratio, (ratio between STT peak to peak amplitude and root mean square value of background noise >20Hz), were considered for analysis and were aligned with respect to the QRS complex by Woody’s method (2). A window of 180ms centered on the mean energy of the QRS and a 500ms window aligned to the STT onset were computed in all 8 leads per participant. Average QRS and STT waveforms were then computed.

## Biomarkers extraction

ECG measures reported in the text and tables are from the 12-lead resting Burdick digital ECG (3), except for the following biomarkers, which were computed from the Holter averaged beat:

- QRS amplitude, defined as the absolute value of the difference between maximum and minimum of the QRS complex values
- the maximum ascending/descending or descending/ascending slopes of the QRS defined as the first ascending and descending slopes depending on the QRS complex pattern
- the percentage of the S wave duration with respect to QRS duration
- STT segment amplitude, defined as the absolute value of the difference between maximum and minimum of the STT segment values
- STT segment elevation, defined as the difference between the beginning of the QRS complex and the beginning of the T wave
- JTc interval, defined as the duration between the end of the QRS (J point) and the end of the T wave, corrected with the Bazett’s formula in each lead
- T peak to T end interval, defined as the duration between the peak of the T wave and the end of the T wave in each lead
- T wave inversion, defined as a negative inflexion of at least 0.1mV in lead I, II or V3-6.


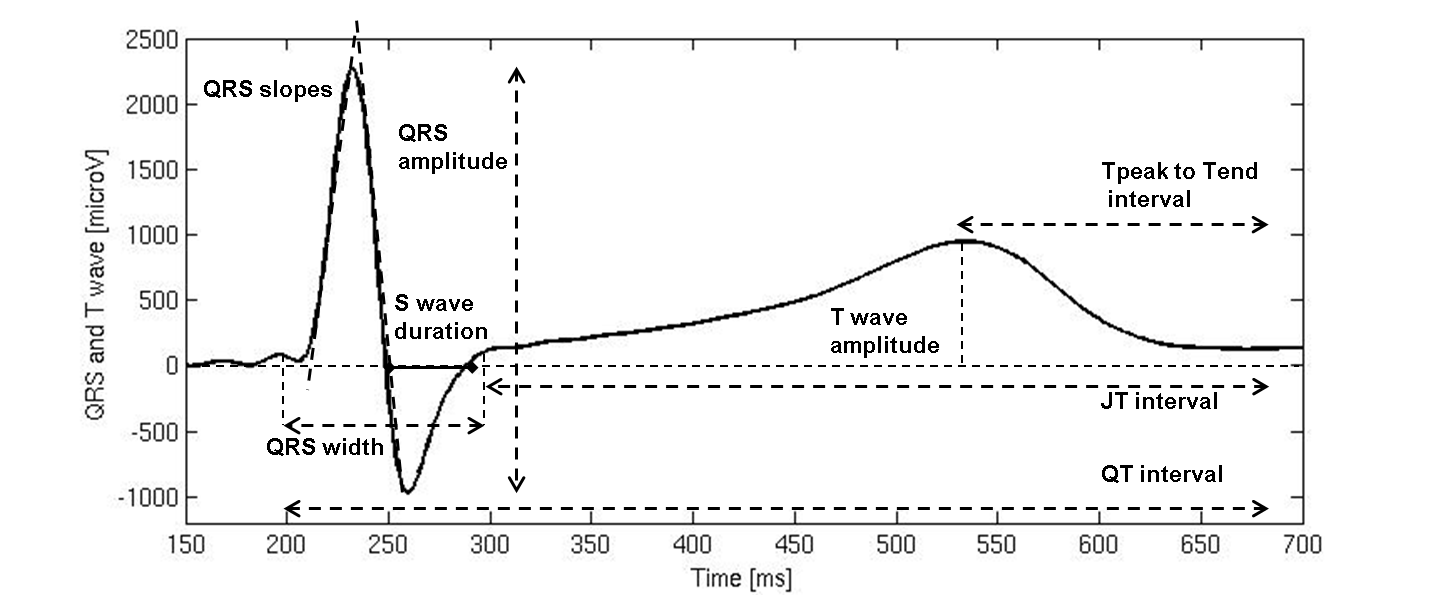


**Figure S1**: Morphological biomarkers extracted from the averaged Holter QRS and T wave

## Hermite transform

The Hermite transform is well known in the literature for its ability to reconstruct the QRS complex (4).

$\forall x\mathbb{\in R},\forall n\mathbb{\in N},\forall\sigma\in\mathbb{N}^{*}, \Psi_{n}\left( x,\sigma\right)={\left( {\sigma2}^{n}n!\sqrt{\pi} \right)^{-\frac{1}{2}}e^{\frac{-x^{2}}{2\sigma^{2}}}H}_{n}\left( \frac{x}{\sigma} \right)$ Equation 1

where $n$ is the order of the Hermite function $\Psi$ and of the associated Hermite polynomial $H$, and $\sigma$ the width parameter


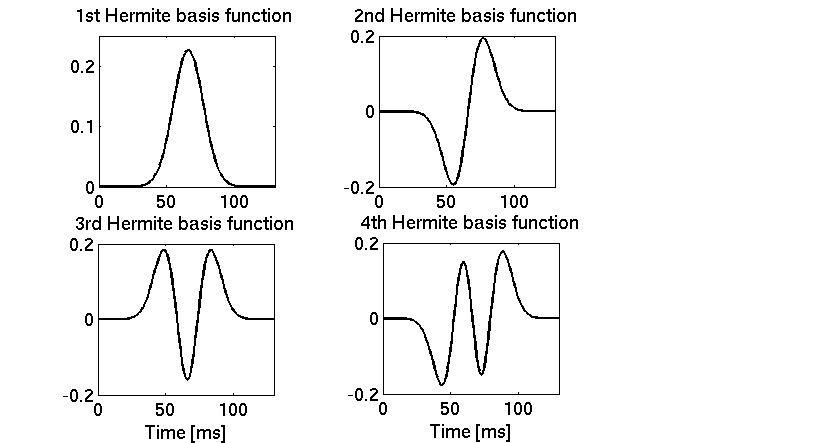


**Figure S2**: First four Hermite basis functions

This transform enables to denoise the signal and provides a more compact representation of the QRS complex (4). In our implementation, QRS was expressed as a linear combination of the first four Hermite bases and a reconstruction error (Equation 2). The width of the Hermite functions was computed for each subject by minimizing the fitting error defined as the root mean square of the difference between the reconstructed and original QRS.

$\forall t\in\left[ 1,2,\ldots,T \right], \hat{x}\left( t \right)=\sum_{n=1}^{4} w_{n}b_{n}\left( t \right)+v(t)$ Equation 2

where $\hat{x}(t)$ is the reconstructed QRS signal, $b_{n}(t)$ the Hermite function of order $n$, $t$ the time samples from $T$ = 1 to 140ms, $w_{n}$ the Hermite coefficients in the linear combination and $v(t)$ the reconstruction error.

## Dimensionality reduction for clustering

The 7 features obtained from feature selection were reduced to two dimensions using Laplacian eigenmaps dimensionality reduction by preserving the local geometrical properties, by computing the eigenvalues and eigenvectors of the graph Laplacian generalized eigenvector problem (6) (Equation 5).

$Ly=\lambda Dy$ Equation 5

with $y$ and $\lambda$ the eigenvectors and eigenvalues of the eigenvector problem, D a diagonal weight matrix computed by constructing the adjacency graph, W such that $D_{ii}=\sum_{j} W_{ji}$, and $L=D-W$ the Laplacian matrix. This process reduced the number of variables under consideration and obtained the principal variables. When T wave biomarkers were also included, three features from the QRS and three from the T wave, were selected and then reduced to two dimensions.

## Statistical analysis

Circular statistics were used for QRS and T wave axis since the angles “wrap” around a circle (i.e. an axis of 180º is equivalent to -180º). This angular data was expressed as circular mean ± circular standard deviation and compared using Watson-Williams F test (8).

# Results

## Hermite coefficients for clustering with QRS morphology alone


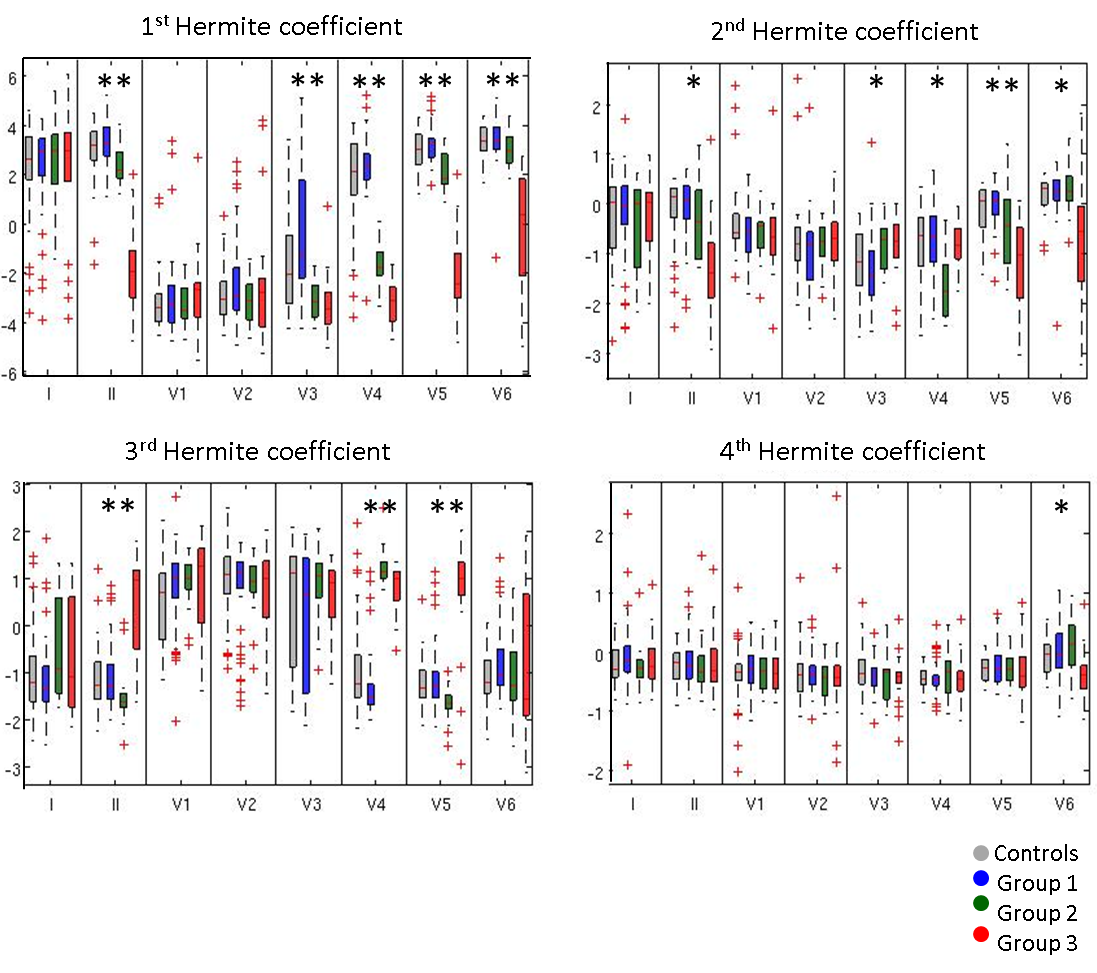


**Figure S3**: Comparison of the four Hermite coefficients in leads I-II, V1-6 for the three QRS-based HCM groups. Controls are shown for visual comparison but were not included in Kruskal-Wallis one-way analysis of variance. Mean, standard deviation and interquartile range; p-values are Bonferroni-corrected for multiple comparisons (*p-value<0.001, **p-value<1×10^-6^).

## ECG and clinical features for the three HCM groups from QRS clustering alone

**Table S1:** ECG features for QRS-based HCM groups

[mean ± standard deviation, or number of patients (%)]

|  | **Group 1**  (n=44) | **Group 2**  (n=19) | **Group 3**  (n=22) | **p value**  (group comparison) |
| --- | --- | --- | --- | --- |
| Heart rate, bpm | 58 ± 9 | 59 ± 13 | 55 ± 10 | 0.46 |
| QRS axis, ^o^ | 30 ± 33 ***^b^*** | 29 ± 34 ***^c^*** | -37 ± 28 | **1×10^-11^** |
| QRS duration, ms | 98 ± 17 | 96 ± 16 | 102 ± 13 | 0.13 |
| QRS amplitude, mV | 1876 ± 702 | 1807 ± 588 | 1737 ±613 | 0.88 |
| QRS ascending slope | 94 ± 34 | 92 ± 34 | 76 ± 23 | 0.10 |
| QRS descending slope | -149 ± 58 | -155 ± 60 | -149 ± 56 | 0.95 |
| Pathological Q waves | 3 (7)^*^ | 8 (42) | 9 (41) | **0.0003** |
| T wave axis, ^o^ | 83 ± 60 | 49 ± 47 | 68 ± 41 | 0.15 |
| Abnormal T axis | 17 (41) | 6 (33) | 5 (25) | 0.50 |
| T amplitude, mV | 107 ± 281 | 219 ±209 | 258 ±214 | **0.14** |
| T wave inversion | 20 (46)^*^ | 4 (21) | 1 (5) | **0.001** |
| Giant T wave inversion | 5 (11) | 1 (5) | 0 | 0.29 |
| T peak to T end interval, ms | 85 ± 19 | 86 ± 16 | 84 ± 19 | 1.00 |
| ST displacement, mV | 11 ± 48 ***^b^*** | 38 ± 51 | 26 ± 49 | **0.004** |
| QTc interval, ms | 442 ± 25 | 429 ± 26 | 443 ± 29 | 0.29 |
| JTc interval, ms | 354 ± 71 | 342 ± 97 | 366±145 | 0.74 |
| **Lead V4** |  |  |  |  |
| R duration V4, ms | 47 ± 11 ***^a,b^*** | 38 ± 10 | 41 ± 17 | **0.0001** |
| R amplitude V4, µv | 1310 ± 706 ***^b^*** | 1307 ± 648 ***^c^*** | 551 ± 433 | **0.00002** |
| S duration V4, ms | 36 ± 17 ***^b^*** | 41 ± 13 | 47 ± 19 | **0.01** |
| S amplitude V4, µv | -568 ± 413 ***^a,b^*** | -1170 ± 570 | -1270 ± 863 | **0.00002** |
| **Lead V6** |  |  |  |  |
| R duration V6, ms | 54 ± 13 ***^b^*** | 51 ± 14 ***^c^*** | 31 ± 18 | **0.000006** |
| R amplitude V6, µv | 1290 ± 696 ***^b^*** | 1227 ± 451 ***^c^*** | 635 ± 455 | **0.00008** |
| S duration V6, ms | 17 ± 18 ***^b^*** | 15 ± 17 ***^c^*** | 35 ± 19 | **0.0004** |
| S amplitude V6, µv | -118 ± 148 ***^b^*** | -122 ± 190 ***^c^*** | -501 ± 460 | **0.00006** |

***^a^*** Group 1 *vs* 2, ***^b^*** 1 *vs* 3, ***^c^*** 2 *vs* 3, p<0.05 on post hoc pairwise comparisons (p-values multiplied by 3 for Bonferroni adjustment of 3 tests).

* p<0.05 on post hoc contingency table analysis (p-values multiplied by 6 for Bonferroni adjustment of 3**×**2 combinations).

## Representative standard 12-lead resting ECG for the four ECG phenotypes

**Group 1A**

**
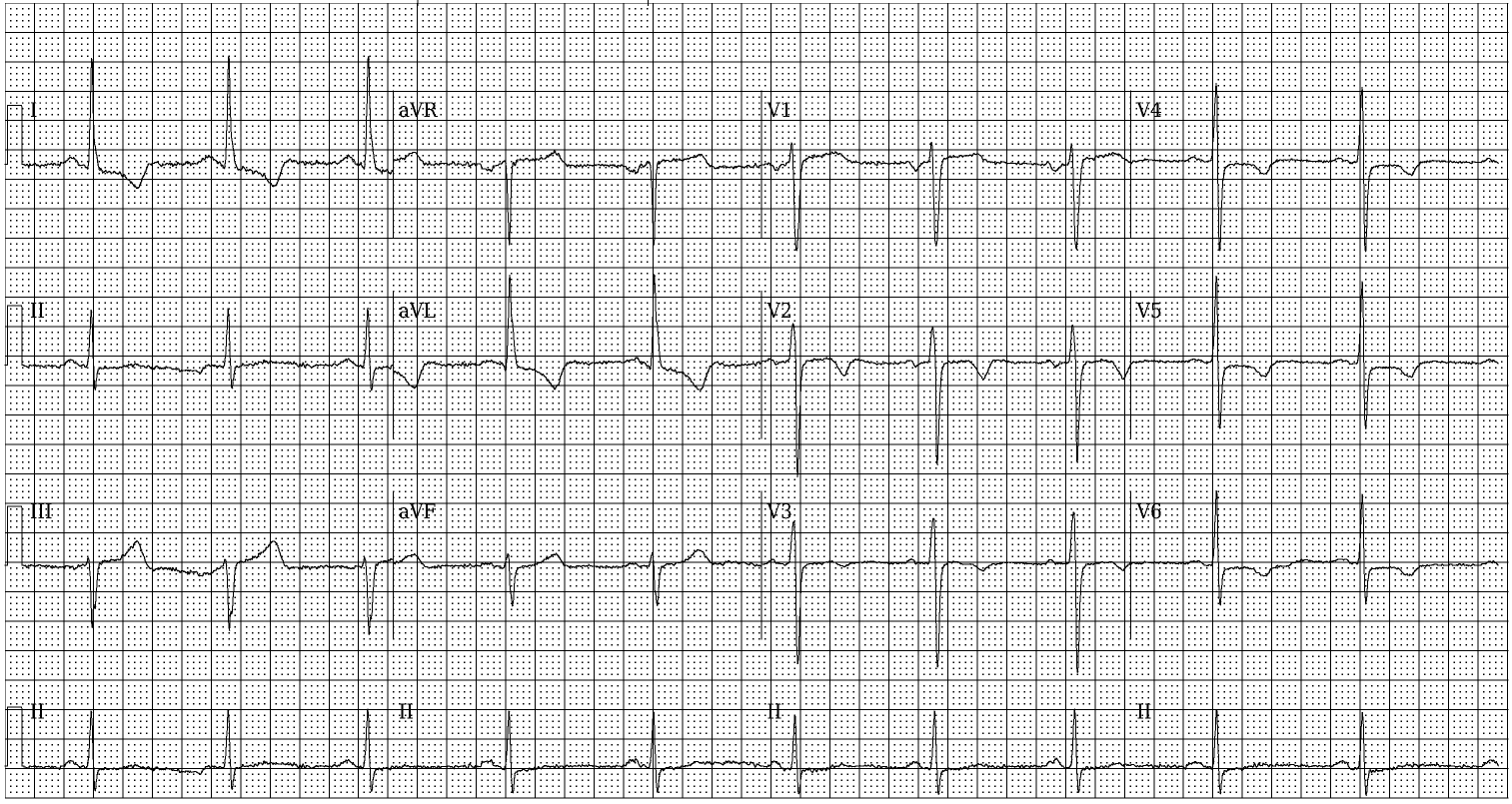
**

**Group 1B**

**
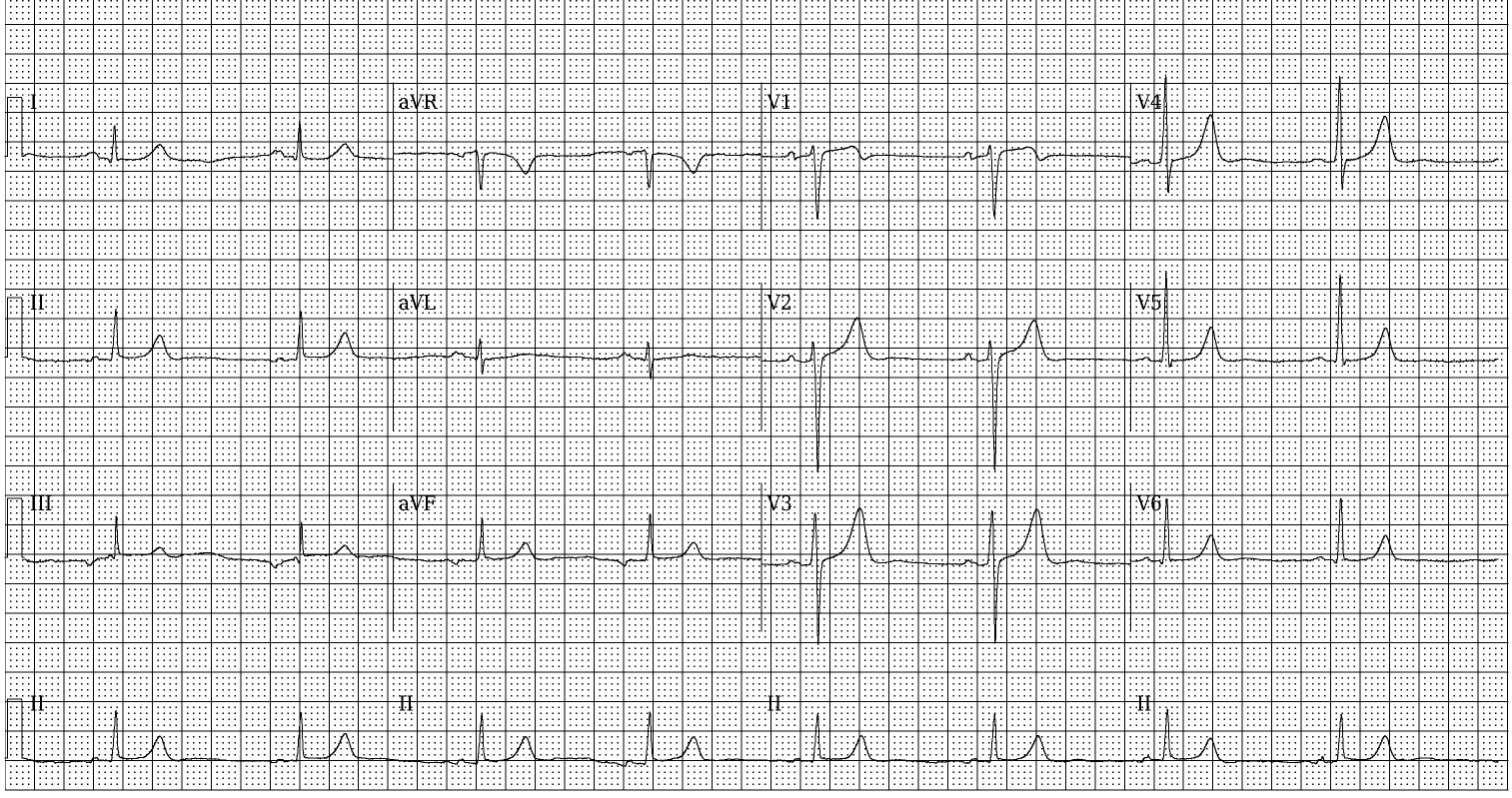
**

**Group 2**

**
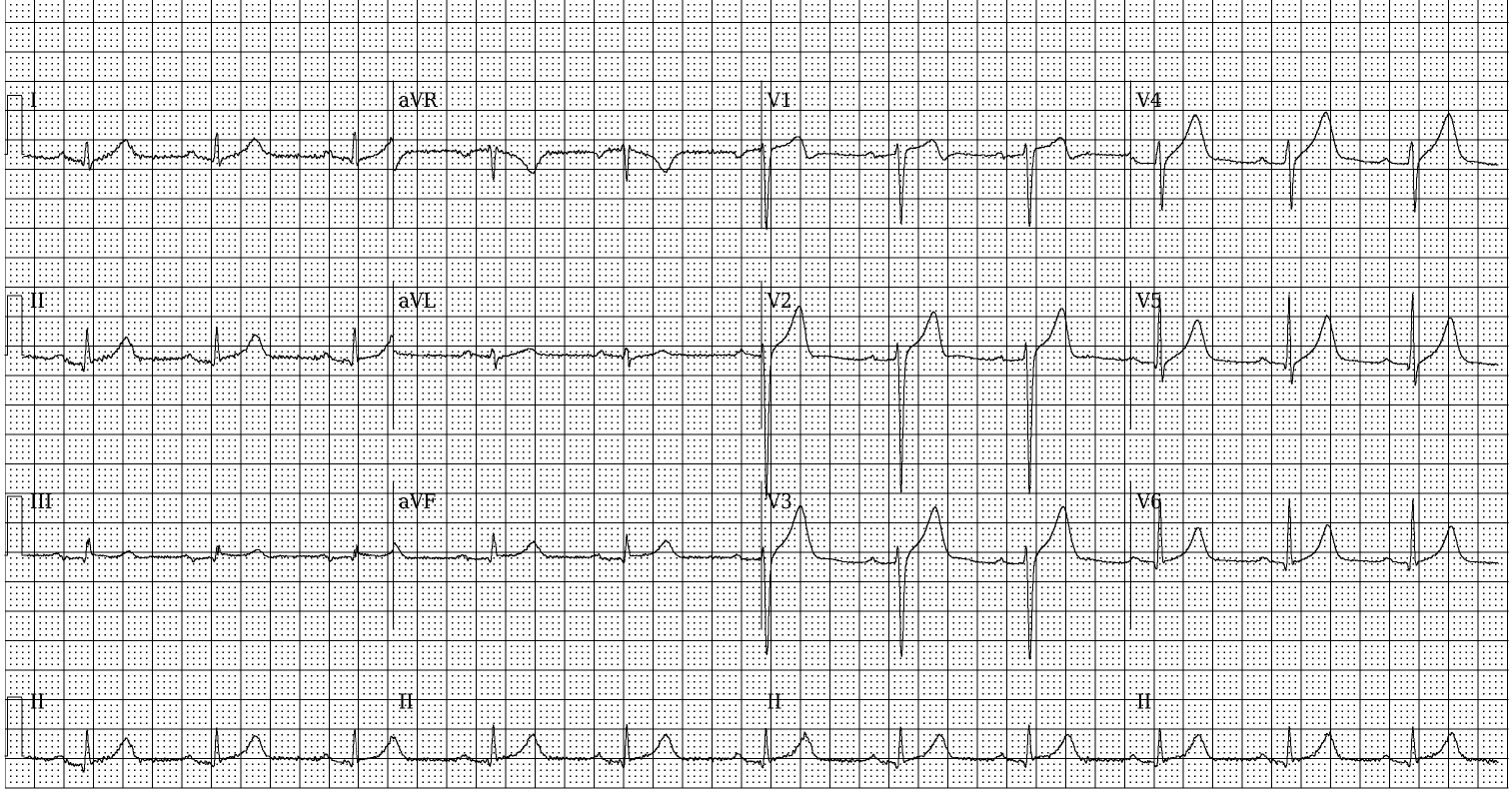
**

**Group 3**

**
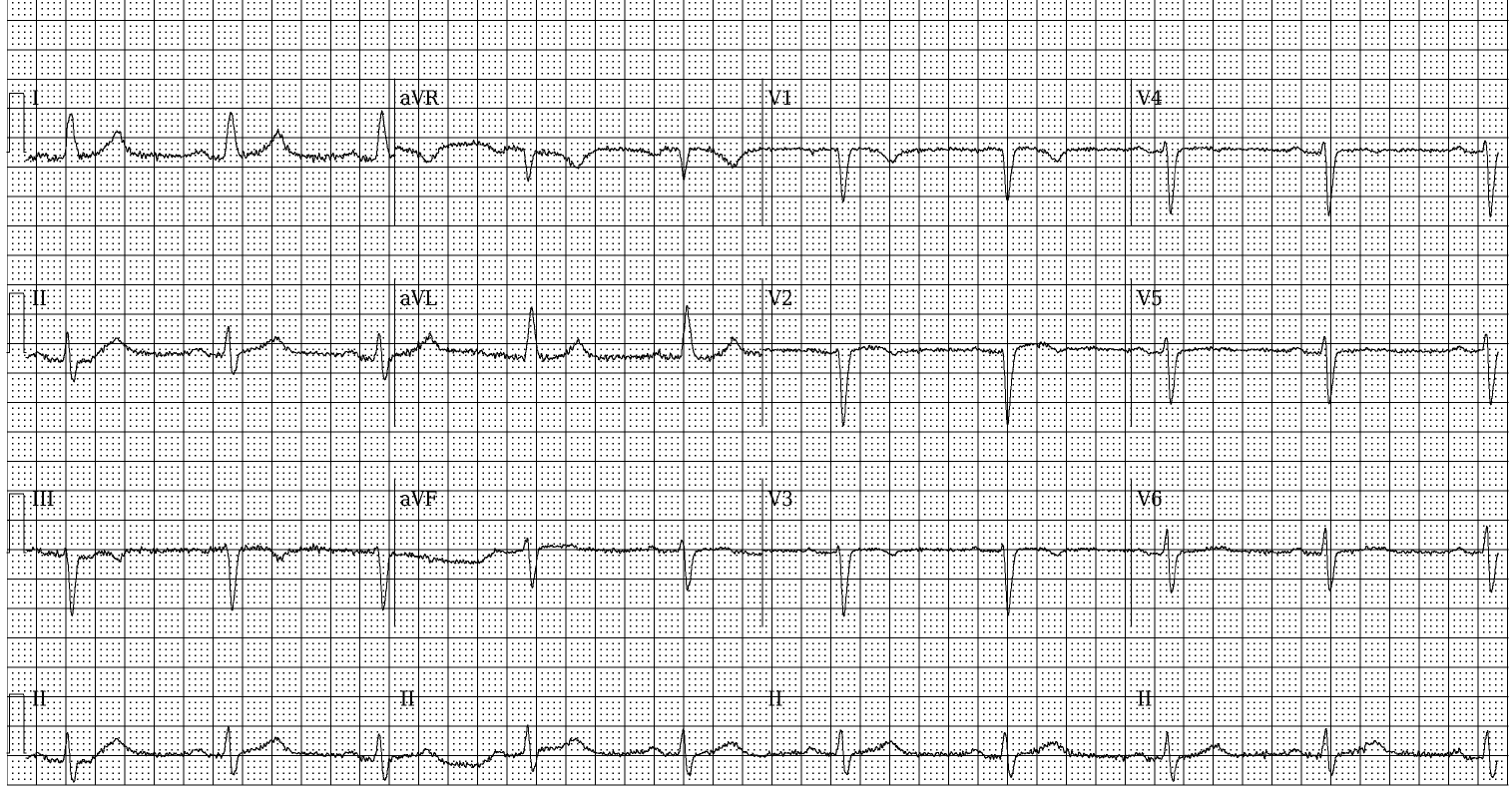
**

## Repeat combined QRS and T wave clustering with the exclusion of gene positive HCM with normal wall thickness (G+LVH-)

In this paper, we consider G+LVH- patients as a cohort of HCM patients consistent with their clinical definition of either presence of a pathogenic genetic mutation or, in the absence of an identified mutation, LVH (≥15mm) not originating from another cause. The consideration of G+LVH- patients allows to evaluate, firstly, whether, other structural and electrophysiological abnormalities in the absence of hypertrophy may yield ECG abnormalities. For example, 5 of the 9 G+LVH- patients displayed LVH on ECG voltage criteria. It is a strength of our study that our method clusters these patients in Group 1B based solely on ECG features, with no prior information on their hypertrophy morphology. To further investigate their influence in the phenotypic classification, we performed our clustering analysis removing the G+LVH- patients. We obtained the same four remaining groups as the one defined before, with the same differences in QRS and T wave morphologies, and Group 1A still identified as higher risk than other subgroups (Table S3 below).

**Table S2: Key characteristics of HCM phenotypes obtained from combined QRS and T wave clustering following the exclusion of gene positive HCM with normal wall thickness.**

[mean ± standard deviation, median (range) or number of patients (%)]

|  | **Group 1A**  (n=20) | | **Group 1B**  (n=17) | | **Group 2**  (n=19) | **Group 3**  (n=20) | **p value**  (group comparison) |
| --- | --- | --- | --- | --- | --- | --- | --- |
| **HCM Risk-SCD score**, % | | 3.4 (2-11) *^a,b,c^* | | 1.9 (1-4) | 2.1 (1-6) | 2.2 (1-9) | **0.004** |
| **Hypertrophy** |  | |  | |  |  |  |
| LV mass index, g/m^2^ | 90.1±27.3 *^b^* | | 69.8±23.1 | | 67.0±18.1 | 71.4±26.2 | **0.02** |
| Max LV wall, mm | 22.0±4.1 | | 19.5±3.6 | | 19.4±4.9 | 21.2±5.8 | 0.24 |
| **Hypertrophy morphology** | | |  | |  |  | **4x10^-5^** |
| Septal LVH | 7 (35) ^*^ | | 16 (94) | | 17 (89) | 18 (90) |  |
| Apical LVH | 2 (10) | | 0 | | 1 (5) | 1 (5) |  |
| Mixed septal & apical LVH | | 11 (55) ^*^ | 1(6) | | 1(5) | 1(5) |  |

HCM, hypertrophic cardiomyopathy; SCD, sudden cardiac death; LV, left ventricular; LVH, left ventricular hypertrophy.
***^a^*** Group 1A *vs* 1B, ***^b^*** 1A *vs* 2, ***^c^*** 1A *vs* 3, p<0.05 on post hoc pairwise comparisons
* p<0.05 on post hoc contingency table analysis

## References

1. O’Mahony C, Jichi F, Pavlou M, Monserrat L, Anastasakis A, Rapezzi C, et al. A novel clinical risk prediction model for sudden cardiac death in hypertrophic cardiomyopathy (HCM Risk-SCD). Eur Heart J. 2014 Aug 7;35(30):2010–20.

2. Woody CD. Characteristics of an adaptive filter for the analysis of variable latency neuroelectric signals. Med Amp Biol Eng. 1967;5(6):539–54.

3. Macfarlane PW, Devine B, Latif S, McLaughlin S, Shoat DB, Watts MP. Methodology of ECG interpretation in the Glasgow program. Methods Inf Med. 1990 Sep;29(4):354–61.

4. Laguna P, Jane R, Olmos S, Thakor NV, Rix H, Caminal P. Adaptive estimation of QRS complex wave features of ECG signal by the Hermite model. Med Biol Eng Comput. 1996 Jan;34(1):58–68.

5. Cai D, Zhang C, He X. Unsupervised Feature Selection for Multi-cluster Data. In: Proceedings of the 16th ACM SIGKDD International Conference on Knowledge Discovery and Data Mining [Internet]. New York, NY, USA: ACM; 2010 [cited 2016 Jun 10]. p. 333–342. (KDD ’10). Available from: http://doi.acm.org/10.1145/1835804.1835848

6. Belkin M, Niyogi P. Laplacian Eigenmaps for Dimensionality Reduction and Data Representation. Neural Comput. 2003 Jun;15(6):1373–1396.

7. Ester M, Kriegel H, S J, Xu X. A density-based algorithm for discovering clusters in large spatial databases with noise. In AAAI Press; 1996. p. 226–231.

8. Philipp B. CircStat: a MATLAB toolbox for circular statistics. ResearchGate [Internet]. 2009 Sep 1 [cited 2016 Nov 30];31(10). Available from: https://www.researchgate.net/publication/38105304_CircStat_a_MATLAB_toolbox_for_circular_statistics
